# Morphological, Pathological and Genetic Diversity of the *Colletotrichum* Species, Pathogenic on Solanaceous Vegetable Crops in Bulgaria

**DOI:** 10.3390/jof8111123

**Published:** 2022-10-25

**Authors:** Vasilissa Manova, Zornitsa Stoyanova, Rossitza Rodeva, Irina Boycheva, Helena Korpelainen, Eero Vesterinen, Helena Wirta, Georgi Bonchev

**Affiliations:** 1 Laboratory of Genome Dynamics and Stability, Institute of Plant Physiology and Genetics, Bulgarian Academy of Sciences, 1113 Sofia, Bulgaria; 2 Laboratory of Applied Genetics and Plant Biotechnologies, Institute of Plant Physiology and Genetics, Bulgarian Academy of Sciences, 1113 Sofia, Bulgaria; 3 Department of Agricultural Sciences, University of Helsinki, P.O. Box 27, 00014 Helsinki, Finland; 4 Department of Biology, University of Turku, P.O. Box 772, 00074 Turku, Finland

**Keywords:** phytopathogenic fungi, *Colletotrichum nymphaeae*, *Colletotrichum godetiae*, *Colletotrichum salicis*, *Colletotrichum cigarro*, multilocus DNA barcoding, genotyping

## Abstract

*Colletotrichum* species are among the most devastating plant pathogens in a wide range of hosts. Their accurate identification requires a polyphasic approach, including geographical, ecological, morphological, and genetic data. Solanaceous crops are of significant economic importance for Bulgarian agriculture. *Colletotrichum*-associated diseases pose a serious threat to the yield and quality of production but are still largely unexplored. The aim of this study was to identify and characterize 26 pathogenic *Colletotrichum* isolates that threaten solanaceous crops based on morphological, pathogenic, and molecular data. DNA barcodes enabled the discrimination of three main taxonomic groups: *C.* *acutatum*, *C.* *gloeosporioides*, and *C.* *coccodes*. Three different species of acutatum complex (*C. nymphaeae*, *C. godetiae*, and *C. salicis*) and *C. cigarro* of the gloeosporioides complex were associated with fruit anthracnose in peppers and tomatoes. The *C. coccodes* group was divided in two clades: *C. nigrum*, isolated predominantly from fruits, and *C. coccodes*, isolated mainly from roots. Only *C. salicis* and *C. cigarro* produced sexual morphs. The species *C. godetiae*, *C. salicis*, and *C. cigarro* have not previously been reported in Bulgaria. Our results enrich the knowledge of the biodiversity and specific features of *Colletotrichum* species, which are pathogenic to solanaceous hosts, and may serve as a scientific platform for efficient disease control and resistance breeding.

## 1. Introduction

Species belonging to the genus *Colletotrichum* are among the most important plant pathogens worldwide from both a scientific and an economic point of view. They affect a wide range of host plants, causing various diseases to all plant organs, with anthracnose as the main devastating disease damaging the aboveground parts of plants, such as fruits, leaves, flowers, and stems [1]. Symptoms of anthracnose fruit rot frequently develop during the postharvest period, not only causing enormous losses in production quality but also possibly having direct implications for human health [2]. Although plants are the main hosts of *Colletotrichum* species, recent studies have shown that insects, animals, and even humans might be infected [3,4]. Therefore, the fight against *Colletotrichum* phytopathogens is of great importance for sustainable crop production and global food safety [5]. Effective tools for accurate species identification and characterization are required to obtain insight into the biology of the genus, the specificity of pathogen–host interactions, and detailed mapping of species distribution. This would allow for the development of strategies for the control of diseases caused by *Colletotrichum* species. [6]. The taxonomy of the genus *Colletotrichum* is quite complex. Distinguishing between species based only on morphological and cultural characteristics is difficult and requires high-level expertise, yet this approach is often unreliable [7,8]. Better classification of the genus *Colletotrichum* requires a combined approach based on molecular genetic analyses and morphological, ecological, and geographical data [9].

Ribosomal sequences were the first DNA regions utilized to discriminate between different *Colletotrichum* species due to the variability of the internal transcribed spacer (ITS), allowing for the construction of species-specific primer combinations [10,11]. Recent developments of new molecular technologies, such as DNA barcoding, have greatly improved the understanding of fungal systematics and *Colletotrichum* taxonomy [12,13]. The progressive accumulation of new molecular evidence, however, brought along substantial revisions of *Colletotricum* systematics [14]. Nowadays, the leading approach utilized to characterize *Colletotrichum* species is based on sequencing data, although classical characteristics are still employed where possible. *Colletotrichum* species are organized into complexes, and the latest research recognizes 14 complexes with large phenotypic and genetic differences between and within them [15,16]. The correct identification of species within a complex is critical for effective disease management, as *Colletotrichum* species frequently show selectivity and variations in their fungicidal sensitivity, as well as species-associated pathogenic potential [17,18].

The identification of species in the genus *Colletotrichum* based on molecular tools is still challenging [19,20]. It is now widely recognized that the ITS region, which is the official fungal barcode, does not work as perfectly for the genus *Colletotrichum* as for some other fungi. Therefore, the misidentification of *Colletotrichum* isolates is not uncommon if based only on one gene region [21,22,23]. Additional barcodes based on other gene regions, such as ACT (actin), TUB2 (β-tubulin), GAPDH (glyceraldehyde-3-phosphate dehydrogenase), CHS-1 (chitin synthase), CAL (calmodulin), and GS (glutamine synthetase) have been applied with variable levels of success [24,25,26]. Intergenic sequences, such as APMAT or APN2/MAT-IGS and GAP-IGS, have also been considered as promising candidates [27,28,29]. However, genomic regions vary greatly in their resolution for individual *Colletotrichum* species complexes, such as APMAT, which is highly informative for species in the gloeosporioides complex but does not perform as well in other complexes. Several studies have demonstrated the power and limitations of ITS and protein coding sequences to distinguish *Colletotrichum* species between and within the species complexes [28,30,31,32,33,34,35]. All these studies reveal, however, that there is no universal barcode sequence or a combination of DNA barcodes suitable for distinguishing species in all groups of the genus, although some of them seem to work better than others for certain species complexes. Therefore, a consensus has emerged that multilocus analyses are needed to accurately define *Colletotrichum* species, although including more sequence regions might complicate data interpretation [1,29]. GAPDH and TUB2 have emerged as the superior barcodes in most of the *Colletotrichum* complexes, but they still need to be complemented with complex specific barcodes for correct species delimitation [29].

The gloeosporioides species complex comprises important phytopathogenic species that cause significant losses in crop production, both in the field and during the postharvest preservation of many fruits. This complex includes many cryptic species that frequently go undetected, even by experienced plant pathologists, if no molecular data are available. Secondary barcodes based on various genomic regions, such as GAPDH, GS, and APN2/MAT are essential for further improvement of taxa discrimination that was initially undertaken based on ITS sequences [23,27]. Thus, in many cases, the multilocus barcoding approach is the only method that allows for consistent species delimitation within the gloeosporioides complex [36].

The acutatum species complex includes taxa which are highly variable phenotypically; they show a very broad host range in plants, and some of them may favor specific hosts or geographic areas. For others, meanwhile, the host choice may even go beyond the plant kingdom [37,38]. These pathogens are known to be especially destructive for fruits including strawberries and apples, leading to enormous losses in fruit production. Differentiating between *C. gloeosporioides* and *C. acutatum* species can be difficult even when based on well-recognized morphological features. Consequently, a barcoding analysis based on six genes was performed by Damm et al. (2012) to correctly place a number of strains, classically identified in the past as *C. gloeosporioides*, into the *C. acutatum* group. TUB2 and GAPDH were found to be the best working barcodes, able to differentiate all subclades in the acutatum species complex, whereas the worst performing marker regions were ITS and CHS-1 [37,38].

*C. coccodes* is a well-established pathogen on a variety of cultivated plants, although it can cause problems only under suboptimal growth or in plants that are already diseased [39]. *C. coccodes* species have been classified into different vegetative compatibility groups (VCG) based on variations in their morphology and aggressiveness, suggesting high genetic variability, which was later confirmed by molecular data [40]. The genetic similarity between *C. coccodes* isolates within a VCG was found to be greater than that observed between different VCGs [41]. This species seems to be a distinct lineage among the genus *Colletotrichum*, as inferred from a multilocus phylogenetic analysis. In addition, sequence data differentiated two clades within this species—*C. coccodes* and *C. nigrum*. The latter was, until recently, considered questionable [42,43], but is now recognized as a separate species [38]. Nevertheless, data about the most suitable DNA barcodes able to discriminate these closely related species await further assessment.

Solanaceous crops are of significant economic importance for Bulgarian agriculture, especially the four major vegetables (potatoes, tomatoes, peppers, and eggplants). They are also particularly susceptible to *Colletotrichum*-associated diseases, such as black dot in potatoes and anthracnose in tomato, pepper, and eggplant fruits. The symptoms that are usually seen in ripe and overripe fruits are frequently associated with other infected organs of the mother plant, such as the roots, stems, and leaves. The *Colletotrichum* population that is pathogenic on solanaceous crops in Bulgaria is heterogeneous, involving but not limited to at least three groups of species: *C. acutatum*, *C. coccodes*, and *C. gloeosporioides* [44,45,46,47]. In Bulgaria, *C. acutatum* was first discovered on strawberries [48] and later found on pepper and tomato fruits [49,50]. The “unusual” *Colletotrichum* species isolated from naturally infected pepper fruits in Bulgaria was originally attributed to the gloeosporioides complex, based on the discrimination obtained by species–specific primers [45].

We previously identified *C. coccodes*, *C. acutatum* (*s.l.*), and *C. gloeosporioides* (*s.l.*) as causative agents of anthracnose and root rot diseases on solanaceous crops in Bulgaria, utilizing a combined approach based on morphological, cultural, and pathogenic characteristics and species–specific primer combinations for selective amplification of the ITS regions. However, a detailed genetic characterization of the fungal isolates that identifies individual taxa was not possible using classical PCR analysis. Thus, the exact taxonomical identity and characteristics of the respective isolates are still lacking. To our knowledge, no previous studies employ multilocus DNA barcoding analysis for the molecular characterization of *Colletotrichum* species in Bulgaria. The aim of the current work was to identify and characterize *Colletotrichum* isolates that are pathogenic on solanaceous crops in Bulgaria on the basis of their morphological and pathogenic features, and of molecular data obtained by applying contemporary DNA barcoding technology. A group of 26 *Colletotrichum* isolates obtained from infected fruits and roots, mainly from pepper, tomato, and potato plants, were genotyped with the DNA barcode markers ITS, ACT, EF1a, and TUB2, thus allowing for precise species identification and an analysis of their phylogenetic relationships.

## 2. Materials and Methods

### 2.1. Sampling of Infected Plant Material and Colletotrichum Isolation

Infected plants and plant parts from different solanaceous hosts (pepper, tomato, eggplant, and potato) were provided by three main sources: field trials conducted at the Institute of Plant Physiology and Genetics (IPPG), Sofia, vegetable production areas, mainly in Central and Southern Bulgaria, and materials provided by local farmers.

Isolations were made directly from various structures of the fungus, microsclerotia or acervuli, extruding conidia, or indirectly by cutting small pieces (about 4 mm × 4 mm) of infected plant organs at the border between diseased and healthy tissue, followed by surface sterilization, rinsing with sterile distilled water, drying on sterile filter paper, and placing the materials onto petri dishes containing potato dextrose agar (PDA). The isolates, tentatively recognized as *Colletotrichum*, were sub-cultured onto fresh PDA plates. Monoconidial cultures were obtained by diluting the spore suspension from the initial isolates and spreading a loopful on the surface of water agar plates. After about 18 h incubation at 25 °C in the dark, small pieces of agar with single germinated conidia were cut and transferred onto fresh PDA plates.

### 2.2. Morphological and Cultural Characterization

The inoculum to be studied for the growth and sporulation of isolates was incubated on PDA at 25 °C in the dark for 7 days and sub-cultured on PDA by transferring 6 mm diameter discs of mycelium taken from the periphery of actively growing colonies. The cultures were incubated at 25 °C under a nearby UV light with a 12 h photoperiod. Colony characters were noted after 10 d based on colony color, texture, pigment production, the presence or absence of concentric rings, sectoring, and reproductive morphology (acervuli or the conidial masses; microsclerotia). The colony diameter of every culture was recorded after 7 d cultivation. Microscopic preparations were made in clear lactic acid. Characteristics, such as the shape and color of conidia/ascospores, the presence of septa, etc., were recorded (Olympus BX41 with Olympus Cell^F software for Imaging Life Science Microscopy cell). Photographs were taken, and later the width and length were measured from calibrated pictures with 100 measurements per conidia/ascospores and 30 per microsclerotia. The average length and width were calculated. Pictures were taken using a Canon PowerShot A95 digital camera.

### 2.3. Pathogenicity Assay of Colletotrichum Isolates

The pathogenic properties of all isolates (including those from potatoes) were studied on host (tomato, pepper, eggplant) and non-host (green apple) fruits. Detached healthy fruits were prepared by surface sterilization with 0.05% NaClO, and were then rinsed with sterile distilled water three times and air-dried for a few minutes. After pricking with a thin sterile needle, the host fruits were inoculated with agar disks (d = 8 mm) containing mycelium of the respective isolates. Control fruits were inoculated with sterile PDA disks. The fruits were incubated for 7 days at 25 °C and 100% relative humidity and monitored daily for symptoms. Re-isolations from the infected tissues were made at the end of the experiment. Symptoms were described for the naturally and artificially infected plants or plant organs of the respective hosts. The pathogenicity test with green apple was performed according to the method of Talhinhas et al. (2008) [51]. Detached apple fruits were prepared as described above. Holes with a diameter and depth of 0.5 cm were made, and mycelial disks of the same diameter were placed into them. There were four inoculation holes in each apple. Sterile PDA disks were used as a control. The isolates and controls were arranged in a random pattern in 4 replicates, i.e., 16 inoculation holes for each experiment variant. The fruits were incubated at 100% relative humidity and 25 °C for 13 days. Reactions were recorded as spot type and diameter (mm), mycelium formation, and sporulation.

### 2.4. Preparation of Fungal Biomass for Phylogenetic Analysis

The isolates were grown for 7–10 days at 24 ± 2 °C by continuous shaking of an orbital shaker (125 rpm) in Erlenmeyer flasks (300 mL), each containing 100 mL of a liquid culture medium: potato dextrose or glucose casein broth [52]. The inoculum for each flask consisted of 3 disks (d = 4 mm) cut from the periphery of actively growing colonies on the PDA. The mycelium was collected by filtration. It was rinsed several times with sterile distilled water and dried on sterile filter paper, frozen in liquid nitrogen and stored at −65 °C.

A total of 26 *Colletotrichum* isolates selected from our collection were analyzed in the current study, of which 23 were isolated from pepper, potato, tomato, and eggplant. An isolate from banana (imported from Ecuador, bought from a fruit and vegetable store in Bulgaria) was used for comparison with our isolates that belonged to the gloeosporioides complex. Two CBS strains, namely CBS 490.92 and CBS 527.77, were additionally included as references for the acutatum complex and *C. coccodes* group, respectively (Table 1).

Based on our preliminary phenotypic and genotypic (ITS region of ribosomal DNA) characteristics and pathogenicity tests [45,47], the isolates were divided into three groups: Group 1—*C. acutatum* (3 isolates and CBS 490.92 strain), Group 2—*C. gloeosporioides* (4 isolates and *C. musae*) and Group 3—*C. coccodes* (16 isolates and CBS 527.77 strain). Three of the isolates in the third group (Cc7-1, Cc7-2 and Cc26-2) originated from the Republic of North Macedonia, collected during field expeditions. Fungal isolations and further investigations were performed at the IPPG.

### 2.5. DNA Extraction and PCR Amplification

DNA was extracted from fungal mycelium using a DNeasy Plant mini kit (Qiagen, Hilden, Germany) according to the manufacturer’s instructions, with slight modifications concerning lysis temperature and incubation time. Fungal material was ground to a fine powder in liquid nitrogen and incubated in a lysis buffer at room temperature for 30 min, and then subjected to high temperature lysis and RNase treatment at 65 °C for 15 min following the standard protocol. At the end of the extraction, total genomic DNA was eluted in AE buffer (10 mM Tris-HCl, 0.5 mM EDTA, pH 9.0). Three eluates were obtained from each DNA preparation and the concentration and purity of eluted DNAs were assessed using Bio-SpecNano (Shimadzu, Japan) and agarose gel-electrophoresis; thereafter, the respective eluates were combined.

The genetic diversity of the fungal isolates was evaluated based on sequences of four gene regions: the *Internal transcribed spacers* (*ITS*) of ribosomal genes, *Actin* (*ACT*), *Translation elongation factor-1a* (*EF-1a*), and *Beta-Tubulin* (*TUB2*). The primer sequences (synthesized by Microsynth) and PCR conditions that varied among primers are shown in Table 2. PCR amplifications were performed in 25 µL PCR reactions containing 1× HOT FIREPol^®^ Blend Master Mix (Solis BioDyne, Tartu, Estonia), 0.4 µM primers, 1.5–2.5 mM MgCl_2_, and 2 µL template DNA (40–60 ng) under the following amplification conditions: initial denaturation for 12 min at 95 °C, 30–35 cycles, denaturation for 1 min at 95 °C, annealing for 1 min (variable temperature depending on the primer pair), elongation at 72 °C for 1–1.5 min, followed by a final elongation at 72 °C for 5 min. PCR amplification was performed in a Doppio Gradient Thermal cycler (VWR). A non-template reaction (mQ water) was included in each set of PCR reactions as a negative control. PCR products were analysed by neutral agarose gel-electrophoresis. Two or four microliters of each PCR reaction were loaded on 1.2–1.6% agarose gels (SeaKem, Lonza), depending on the length of the expected PCR fragment, along with appropriate molecular markers, stained with GoodView (SBS Genetech, Beijing, China), and run in 1× TAE buffer at 50 V for at least 2 h until the analysed PCR fragments were clearly resolved. Gels were visualized and photographed under UV using a GBOX-CHEMI-XRQ gel documentation system (Syngene, Bangalore, India) and densitometrically analysed using ImageQuant TL7 software (GE Healthcare, Chicago, IL, USA) to determine the specificity, approximate length, and concentration of the amplified PCR products. The rest of each PCR reaction was utilized for sequencing.

### 2.6. DNA Sequencing and Data Analysis

Successful amplicon products for all four gene regions were sequenced in both directions by Microsynth (Göttingen, Germany), using the same primers used for the PCR amplification. The resulting chromatograms were viewed and revised with Chromas 2.6.6. (https://technelysium.com.au/wp/chromas/ (accessed on 1 August 2022)). Candidate DNA barcode sequences for each barcode region were edited and aligned in the software package Molecular Evolutionary Genetics Analysis (MEGA) ver. MEGA11 (http://www.megasoftware.net (accessed on 1 August 2022) [56]) and consensus sequences were subjected to further analyses. The same software was used to calculate the statistical parameters of genetic diversity (total number of sites, number of variable sites, number of parsimony informative sites, singleton sites) for each DNA barcode marker. The taxonomic assignment of analyzed sequences was performed via the Basic Local Alignment Search Tool (BLAST) matches (http://blast.ncbi.nlm.nih.gov/Blast.cgi (accessed on 1 August 2022)) against NCBI’s nucleotide database in GenBank [57]. The four barcode regions of *Colletotrichum coccodes* (CBS 527.77) and *Colletotrichum acutatum* (CBS 490.92), as well as the reference CBS strains, were also sequenced in our study, and were compared to the available sequence data (for CBS 527.77) in the GenBank. Sequences obtained in this study along with the retrieved sequences of the most closely related *Colletotrichum* strains available in GenBank were aligned and manually refined in MEGA ver. MEGA11.

Phylogenetic and molecular evolutionary analyses were conducted using MEGA version 11 [56]. Phylogenetic trees for each individual barcode region were constructed using the UPGMA clustering method [58] and the Kimura 2-parameter model [59]. The stability of the topology of the phylogenetic tree was assessed using the bootstrap method with 500 repetitions [60]. The statistical parameters of genetic diversity within the genus *Colletotrichum* were calculated in MEGA11.

## 3. Results

### 3.1. Morphological and Cultural Characterization of Colletotrichum Isolates

**Figure 1:**
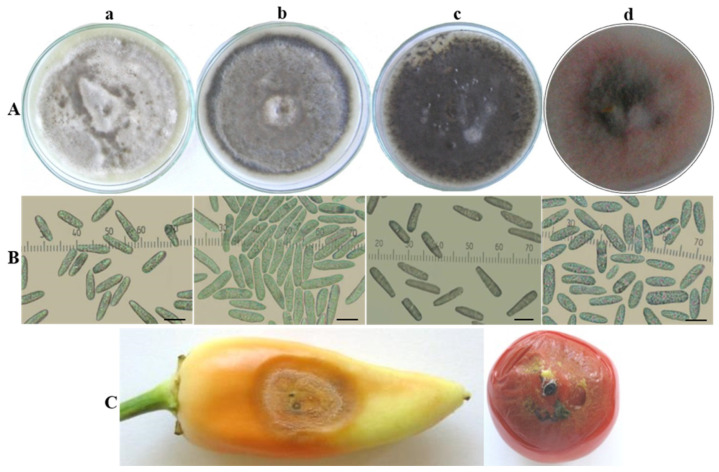
*Colletotrichum acutatum* group. (**A**) Colony morphology and (**B**) conidia of isolates: (**a**) Ca13-1; (**b**) D13-3; (**c**) Ca44-1; (**d**) CBS 490.92. Scale bar = 10 µm. (**C**) Advanced symptoms caused by isolate Ca13-1 on artificially inoculated detached fruits of pepper and tomato plants.

Three isolates referred to as the *acutatum* group (Ca13-1, CaD13-3 and Ca44-1) were included in this study. Ca13-1 was selected from among several isolates obtained from pepper fruits showing the same symptoms and similar morphological and cultural characteristics. CaD13-3 and Ca44-1 were isolated from tomato fruits and expressed some phenotypical differences in symptoms and in culture. The colony morphology and conidia of isolates from the *C. acutatum* group are shown in Figure 1A,B. The isolates developed circular colonies on PDA, which were initially white and compact, with abundant aerial mycelium; only the central part on the underside was dark grey-green. After 10 days of cultivation, the color of the colonies on the upper side changed to light grey (Ca13-1), grey with a lighter center (CaD13-3), or dark grey-green (Ca44-1) and dark grey on the reverse side (Figure 1A(a–c)). Colonies of the CBS strain (490.92) were saffron with beige to grey spots on the upper side, and salmon, pale vinaceous, or olivaceous to purplish grey on the reverse side. The color of the conidial masses was salmon to orange (Figure 1A(d)). The conidia of the studied isolates were aseptate, hyaline, smooth-walled, and straight, with some differences in size and shape (Figure 1B(a–d)). Their dimensions and some specific features are presented in Table 3.

**Figure 2:**
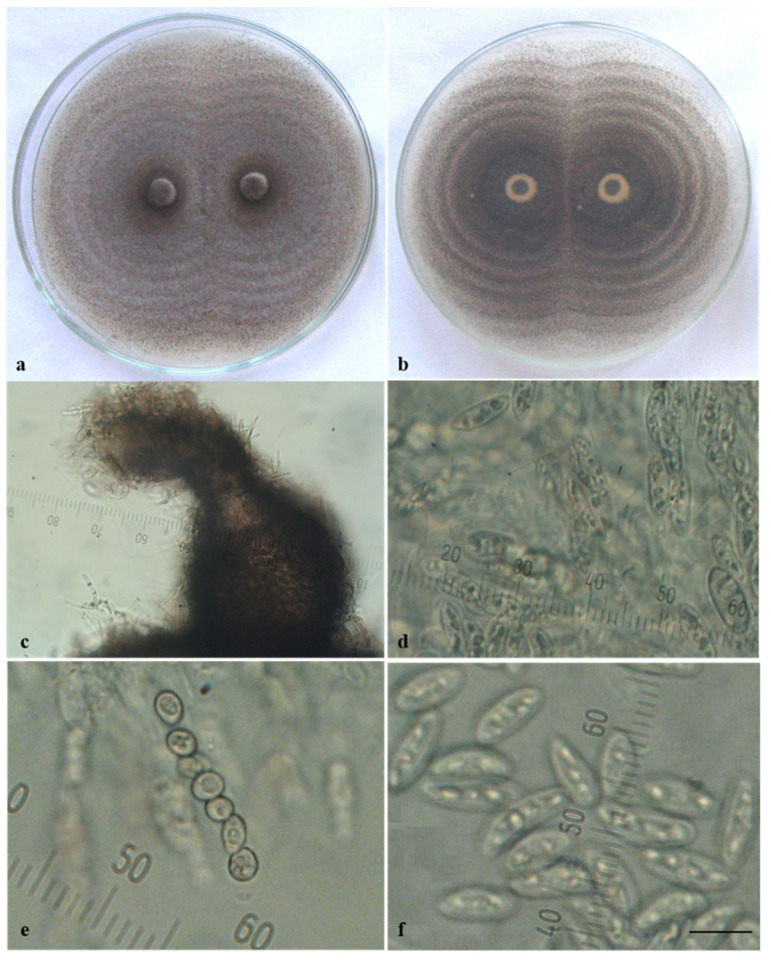
Sexual morph of *Colletotrichum acutatum*. Isolate Ca44-1 produced on PDA by pairing with itself: (**a**,**b**) perithecia arranged in concentric circles (**a**) above; (**b**) reverse; (**c**) perithecium; (**d**) asci with ascospores; (**e**) immature ascospores during division; (**f**) free mature ascospores; scale bar = 10 µm.

Ca44-1 was the only isolate that produced sexual morphs in culture. When it was cultivated individually, predominantly sterile perithecia developed, which were mostly scattered in the colony center (Figure 1A(c)). Fertile structures of the sexual morph (perithecia, asci, and ascospores) were found only in older cultures. It is worth noting that an enormous quantity of fertile perithecia arranged in concentric circles appeared when this isolate was paired with others or especially with itself (Figure 2a,b). They were dark in color, rounded or bottle-shaped, with a diameter of 100–250 µm and a well-developed, slightly curved neck (beak) and ostiole (pore) at the tip, through which ascospores were ejected (Figure 2c). Thin-walled cylindrical asci containing eight ascospores were located in the perithecia (Figure 2d,e). Ascospores were unicellular, hyaline, smooth-walled, straight, and fusiform (Figure 2f), with dimensions (12.2−) 14.0 ± 0.2 (−17.0) × (4.1−) 5.3 ± 0.1 (−6.5) µm.

**Figure 3:**
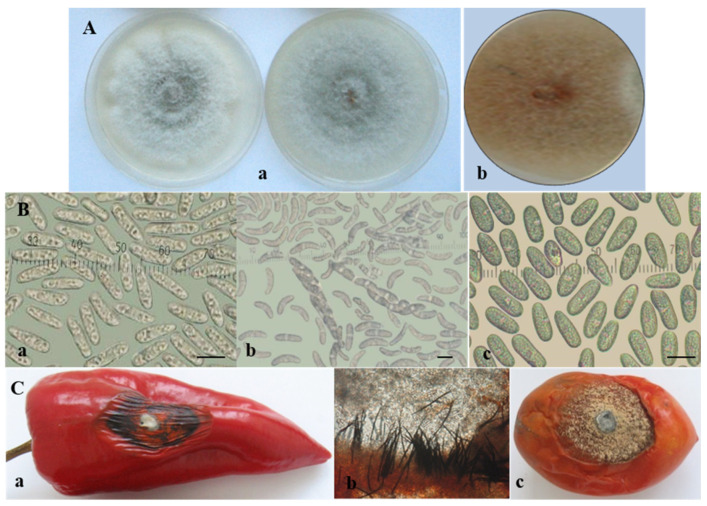
*Colletotrichum gloeosporioides* group. (**A**) Colony morphology of: *C. cigarro* (**a**) and *C. musae* (**b**); (**B**) sporulation: conidia (**a**) and ascospores (**b**) of *C. cigarro;* conidia of *C. musae* (**c**); scale bars = 10 µm; (**C**) advanced symptoms caused by *C. cigarro* (isolate CgD6-1) on artificially inoculated detached fruits of: pepper—(**a**) dark dry wrinkled lesion; (**b**) black sporulating setose acervuli just beneath the skin of the infected fruit (×16) and (**c**) tomato.

Within the gloeosporioides complex, two isolates from pepper, two from tomato, and one from banana were characterized phenotypically. Isolates from this complex were first obtained from naturally infected pepper fruits in Bulgaria in 2010 and described as “unusual” *Colletotrichum* species associated with pepper fruit anthracnose [45]. In 2011, a fungus with similar features was also isolated from tomato plants in Bulgaria. The studied isolates from the *C. gloeosporioides* group had similar characteristics, regardless of whether they originated from pepper or tomato fruits. On PDA they produced light grey circular colonies with a darker center (Figure 3A). Salmon conidial masses appeared among the mycelia. Simultaneously, black fruiting bodies readily emerged in or between the conidial masses and were especially well visible on the rose-ochre reverse side of the agar plate. The conidia of all isolates were straight, hyaline, smooth-walled, aseptate, and cylindrical with two to seven oil globules, and were either rounded at both ends or pointed at one end and rounded at the other (Figure 3B(a)). The conidial size is presented in Table 3. It turned out that black bodies were perithecia with narrow clavate asci measuring (50.8−) 61.7 ± 8.7 (−75.4) µm. Asci contained eight ascospores, which were unicellular, hyaline, smooth-walled, slightly curved to sickle-shaped, ellipsoidal, and slightly narrowed at the edges (Figure 3B(b)), measuring (15.9−) 19.5 ± 1.4 (−21.8) × (3.3−) 4.2 ± 0.6 (−5.5) μm. Sequencing data obtained in the current study clearly identified these isolates as *C. cigarro.* The isolate of *C. musae* (Cm13), also belonging to the gloeosporioides complex, produced only conidia, which were unicellular, hyaline, and cylindrical, with rounded edges (Figure 3B(c)). Sexual morphs were not observed on the natural substrate or in culture.

The *C. coccodes* group was the largest in this study and included seventeen isolates from four solanaceous hosts (pepper, tomato, eggplant, and potato) (Table 1). All isolates produced microsclerotia and that was their essential characteristic, which distinguished them from the other isolates belonging to the *C. acutatum* and *C. gloeosporioides* groups. These were small, dark, globose setose bodies, which emerged in the colony starting from its center and distributing throughout the agar plates, often arranged in concentric circles. On PDA, the sclerotia of all *C. coccodes* isolates showed a tendency to aggregate and reached larger sizes.

**Figure 4:**
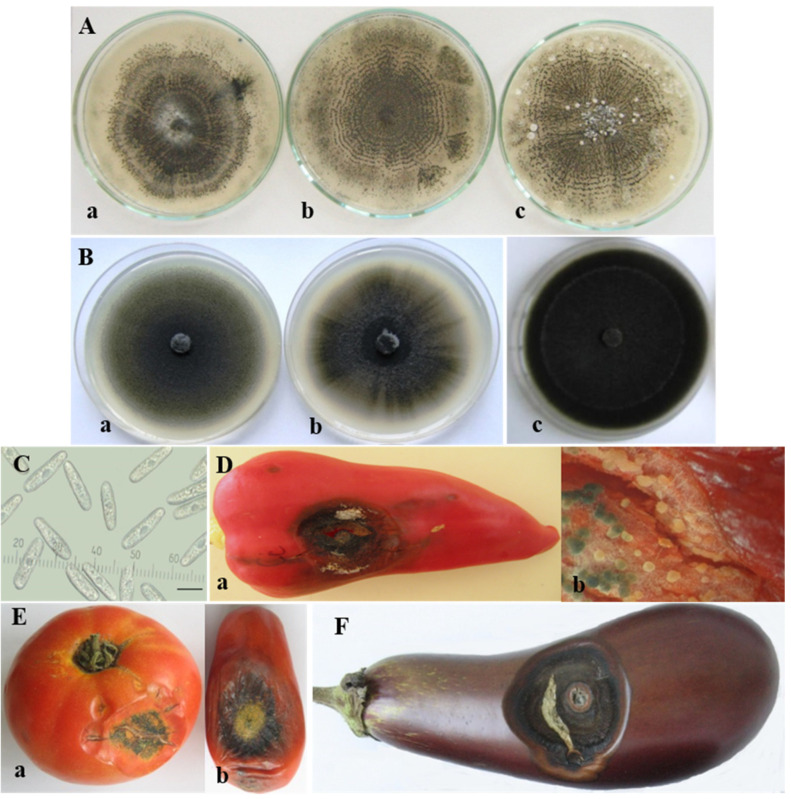
*Colletotrichum coccodes* group. Colony morphology of isolates obtained from: (**A**) fruits of: (**a**) pepper (B8-1); (**b**) tomato (Cc2-1-10); (**c**) eggplant (Cc40-1); (**B**) roots of pepper: (**a**) B12-9; (**b**) K-18 and tomato: (**c**) CBS 527.77; (**C**) conidia; scale bar = 10 µm; (**D**–**F**) advanced symptoms caused by *C. coccodes* (isolate B8-1) on artificially inoculated detached fruits of: (**D**) pepper—(**a**) lesion with microsclerotia on the upper part of the fruit; (**b**) sporulating acervuli inside the fruit beneath the lesion (×16); (**E**) tomato—(**a**) salad variety; (**b**) processing variety; (**F**) eggplant.

The isolates obtained from pepper, tomato, and eggplant fruits developed white circular colonies on PDA and become grey with age, with a narrow pink sporulating periphery and well-defined concentric zones with microsclerotia. The surface was mainly covered by grey creeping mycelium. Unevenly distributed white to light grey tufts of sparse cottony aerial mycelia were occasionally observed (Figure 4A).

The isolates of pepper and tomato roots showed different colony morphology than those of the fruits (Figure 4B). Colonies were greenish grey to dark grey from above, and dark grey to black on the reverse side with more or less pronounced concentric and/or radial zones. As the root rot caused by *C. coccodes* had a more pronounced effect on pepper than on tomato and eggplant, it deserved special attention. A detailed comparative study on isolates of pepper roots and fruits was previously performed by Stoyanova et al. (2013) [61].

The isolates of *C. coccodes* that cause black dot disease in potatoes were described in detail by Rodeva et al. (2016) [46]. Here, it is worth noting that only three of the studied isolates were phenotypically similar to the isolates of *C. coccodes* from roots, of which isolate P5-4 from *S. tuberosum* showed the greatest similarity with the reference strain CBS 527.77 from tomato roots. Isolate P13-2-1, obtained from the potato stolon, exhibited a different phenotype and was morphologically close to the fruit isolates. The colonies were lighter in color. Concentric circles started from the center of the colony and contained a larger number of more aggregated sclerotia, between which the color was pink. The periphery had irregular contours, whitish to pale pink in color.

The conidia of *C. coccodes* appeared as a slimy mass on the colony periphery, where the fungus actively grew with a pink or pale pink color. They were unicellular, hyaline, straight, and cylindrical to fusiform (Figure 4C). The conidial characteristics of the *Colletotrichum coccodes* isolates are presented in Table 3.

### 3.2. Pathogenicity of Colletotrichum Isolates

The pathogenicity of isolates was studied on host (tomato, pepper, eggplant) and non-host (green apple) fruits. All studied *Colletotrichum* isolates were pathogenic for pepper and tomato and exhibited more-or-less similar symptoms on inoculated fruits in the beginning of the infection process. Three days after inoculation (dai), water-soaked circular lesions emerged; these became soft and slightly sunken. About 10 dai, the appearance of the lesions changed. The central parts of the lesions on fruits inoculated by isolates from the *C. acutatum* group was covered by a large number of acervuli without setae, extruding yellowish- to salmon-colored conidial masses (Figure 1C).

The lesions on pepper fruits inoculated by four isolates from the *C. gloeosporioides* group became more dark, dry, and leathery, and the infected tissue—together with the adjacent healthy tissue—began to wrinkle (Figure 3C(a)). Densely ordered small black acervuli with dark setae arranged in concentric circles appeared, starting from the center of the lesion and extending to the periphery of the lesion. They were located just beneath the skin of the infected fruit, which ruptured with the growth of conidiophores, and conidia emerged as salmon-colored slimy masses (Figure 3C(b)). The inoculation of tomato fruits by the same isolates led to the appearance of round, fast-growing spots. The lesions were semi-dry and formed a large number of acervuli with abundant sporulation. The fruits wrinkled and mummified with aging (Figure 3C(c)).

The pathogenicity tests with *C. coccodes* were successfully performed on pepper, tomato, and eggplant fruits. On peppers, fully developed lesions were usually round or slightly elongated, concave, and varied in color from dark red to yellow-brown and black (Figure 4D(a)). At first, setose acervuli extruding conidial masses appeared outside and inside the infected fruits (Figure 4D(b)). Very soon, small dark spherical setose microsclerotia developed from the stromata of acervuli within the infected fruit tissue. In tomatoes (Figure 4E(a,b)) and eggplants (Figure 4F), symptom development was similar. Cracking and splitting of the diseased tissue were common. Successful re-isolations were made from all inoculated fruits.

**Figure 5:**
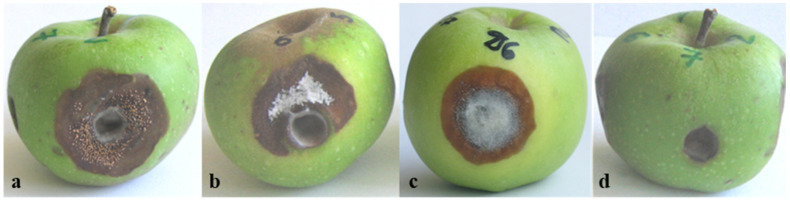
Pathogenicity tests on green apples, conducted according to Talhinhas et al., 2008 [51], performed with different *Colletotrichum* species: (**a**) *C. nymphaeae* (Ca13-1); (**b**) *C. salicis* (Ca44-1); (**c**) *C.* *cigarro* (CgD6-1); (**d**) *C. coccodes* (Cc7-1). Designations of the isolates used for inoculation are shown in parentheses.

Pathogenicity tests performed on green apple fruits with isolates from different *Colletotrichum* species resulted in the development of specific spot types (Figure 5). After inoculation with isolates from the acutatum complex (Ca13-1 and CaD13-3, defined in this study as *C. nymphaeae* and *C. godetiae*, respectively), large brown spots with scattered acervuli developed, tearing the fruit skin and releasing abundant salmon-colored conidial masses. As the lesions caused by both isolates were very similar, only the symptoms on *C. nymphaeae* are shown (Figure 5a). Isolate Ca44-1, also belonging to the acutatum complex, but here referred to as the species *C. salicis*, caused the formation of large brown-to-dark-brown spots, slightly concave, partially covered with light grey mycelium, and bore fewer sporulating acervuli (Figure 5b). Inoculation with four isolates of the gloeosporioides complex (here defined as *C. cigarro*) led to the development of a large round area of necrotic tissues with a dense light grey mycelium in the center and conidial masses emerging from densely arranged small acervuli (Figure 5c). Isolates producing microsclerotia and defined as *C. coccodes* showed the lowest virulence. The necrosis was limited to the inoculation opening, in which a sparse grey mycelium was observed; however, acervuli and microsclerotia did not appear (Figure 5d). Successful re-isolations were made directly from the conidial mass of the acervuli (the *acutatum* and *gloeosporioides* groups) or from the spots around the inoculation openings. The colonies grown after re-isolation were phenotypically identical to those initially used as inoculum.

### 3.3. Genetic Diversity and Phylogenetic Analysis

#### 3.3.1. Efficiency of PCR Amplification and DNA Sequencing

The success rates of PCR amplification and sequence efficiency were measured for all obtained DNA barcodes. In the group of *Colletotrichum* isolates studied, three of the barcodes, namely ITS, ACT, and TUB2, showed 100% amplification and sequencing efficiency among the isolates (Table 4). The EF-1a region was an exception, with an amplification rate of 75%. The short fragment of the EF-1a gene was amplified in all *C. coccodes* isolates and in two isolates from the *C. acutatum* group (Ca44-1 and CBS 490.92), resulting in good quality sequences. The other two *C. acutatum* and all *C. gloeosporioides* isolates lacked any PCR amplification of the EF-1a barcode and were therefore excluded from the analysis. The respective lengths of the PCR fragments were: ~600 bp for the ITS region, ~300 bp for ACT, and ~200 bp (EF-1a) and ~800 bp for the TUB barcode.

#### 3.3.2. Genetic Diversity and Phylogenetic Analyses

Overall, the genetic divergence among the *Colletotrichum* isolates was indicated by the presence of 1–5 bp deletions and SNPs (data not shown). Table 5 shows the parameters of genetic diversity of the studied DNA barcode regions. The total number of sites varied between 242 (EF-1a) and 750 (TUB2). The number of variable sites ranged from 115 for the ITS region to 328 for TUB2. The number of parsimony-informative sites was highest for TUB2.

**Figure 6:**
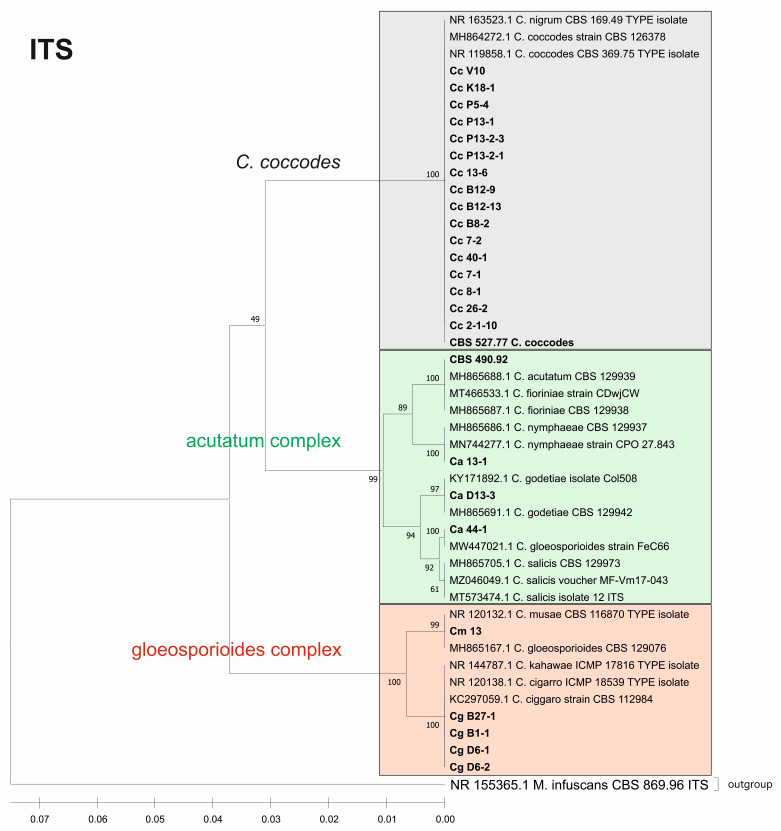
Phylogenetic tree based on the ITS barcode. Genetic diversity and taxonomic assignment of the studied *Colletotrichum* isolates against accessions in the NCBI database and assessed by the DNA barcode for the ITS locus. The *Colletotrichum* clades coccodes, gloeosporioides, and acutatum are depicted by grey, red, and green boxes, respectively. The trees were constructed in MEGA11 using the UPGMA clustering method and the Kimura 2-parameter model. The percentage of replicate trees in which the associated taxa clustered together in the bootstrap test (500 replicates) is shown next to the branches. The tree is drawn to scale, with branch lengths in the same units as those of the evolutionary distances used to infer the phylogenetic tree. All ambiguous positions were removed for each sequence pair (pairwise deletion option). Sequences derived from the NCBI are shown with their accession number and organism name.

**Figure 7:**
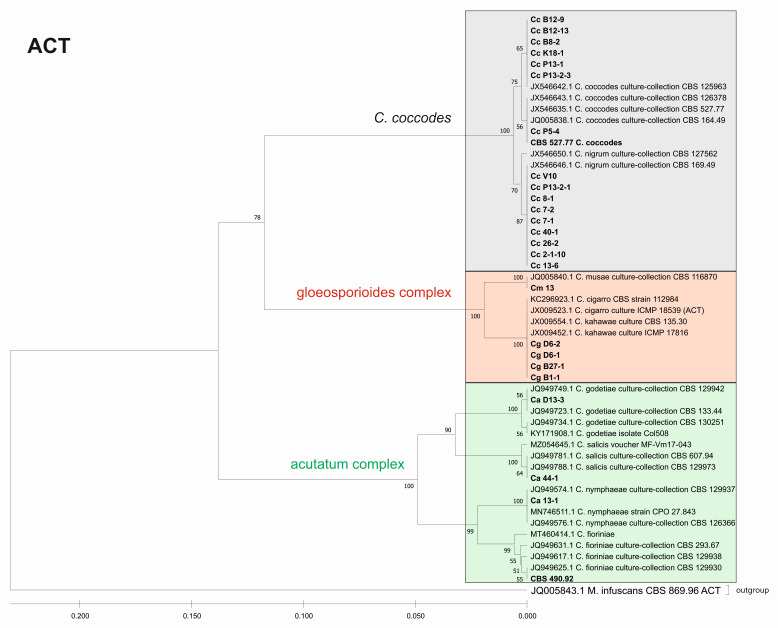
Phylogenetic tree based on ACT barcode. Genetic diversity and taxonomic assignment of the studied *Colletotrichum* isolates against accessions in the NCBI database and assessed by DNA barcode for the actin (ACT) locus. The *Colletotrichum* clades coccodes, gloeosporioides, and acutatum are depicted by grey, red, and green boxes, respectively. The trees were constructed in MEGA11 using the UPGMA clustering method and the Kimura 2-parameter model. The percentage of replicate trees in which the associated taxa clustered together in the bootstrap test (500 replicates) is shown next to the branches. The tree is drawn to scale, with branch lengths in the same units as those of the evolutionary distances used to infer the phylogenetic tree. All ambiguous positions were removed for each sequence pair (pairwise deletion option). Sequences derived from the NCBI are shown with their accession number and organism name.

**Figure 8:**
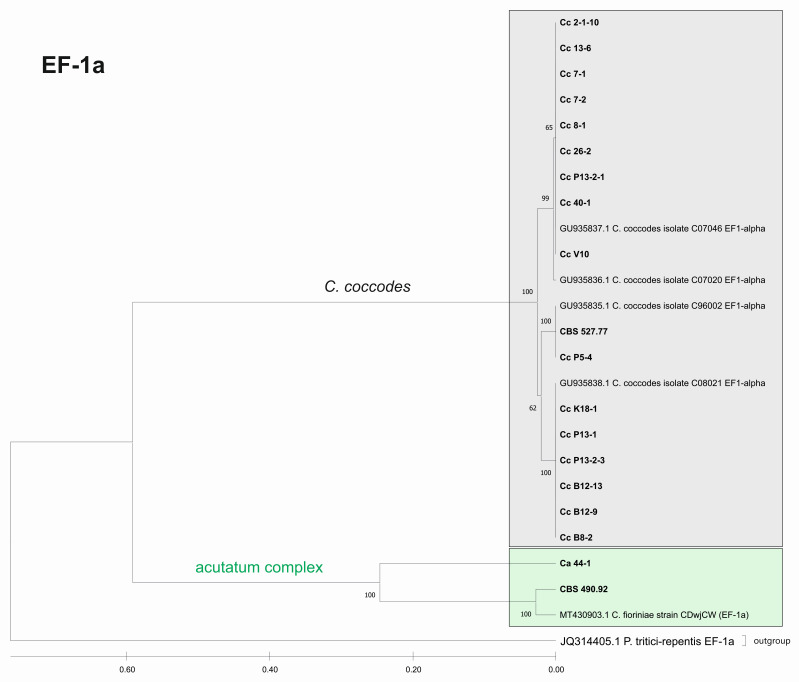
Phylogenetic tree based on the EF-1a barcode. Genetic diversity and taxonomic assignment of studied *Colletotrichum* isolates against accessions in the NCBI database and assessed by the DNA barcode for the locus translation elongation factor 1-alpha (EF-1a). The *Colletotrichum* clades coccodes and acutatum are depicted by grey and green boxes, respectively. The trees were constructed in MEGA11 using the UPGMA clustering method and the Kimura 2-parameter model. The percentage of replicate trees in which the associated taxa clustered together in the bootstrap test (500 replicates) is shown next to the branches. The tree is drawn to scale, with branch lengths in the same units as those of the evolutionary distances used to infer the phylogenetic tree. All ambiguous positions were removed for each sequence pair (pairwise deletion option). Sequences derived from the NCBI are shown with their accession number and organism name.

**Figure 9:**
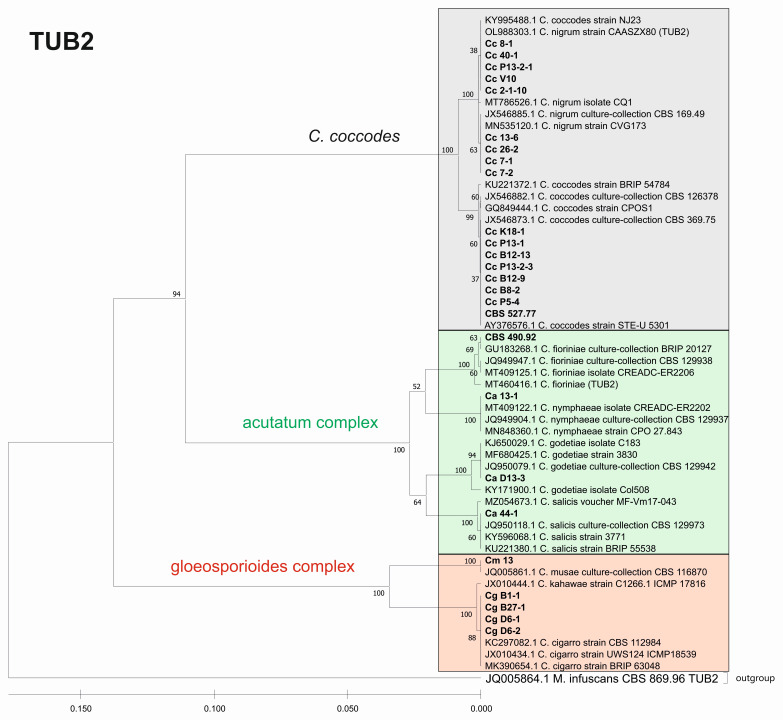
Phylogenetic tree based on TUB2 barcode. Genetic diversity and taxonomic assignment of the studied *Colletotrichum* isolates against accessions in the NCBI database and assessed by DNA barcode for the beta-tubulin (TUB2) locus. The *Colletotrichum* clades coccodes, gloeosporioides, and acutatum are depicted by grey, red, and green boxes, respectively. The trees were constructed in MEGA11 using the UPGMA clustering method and the Kimura 2-parameter model. The percentage of replicate trees in which the associated taxa clustered together in the bootstrap test (500 replicates) is shown next to the branches. The tree is drawn to scale, with branch lengths in the same units as those of the evolutionary distances used to infer the phylogenetic tree. All ambiguous positions were removed for each sequence pair (pairwise deletion option). Sequences derived from the NCBI are shown with their accession number and organism name.

Furthermore, we constructed phylogenetic trees based on the barcode sequences ITS, ACT, TUB2 and EF-1a to infer the genetic distances and taxonomic relationships between the isolates. The taxonomic assignment of the studied isolates to the three *Colletotrichum* groups—*coccodes*, *gloeosporioides* and *acutatum*—was performed against *Colletotrichum* accessions retrieved from the NCBI database. *Monilochaetes infuscans* strain CBS 869.96 was added as an outgroup for the ITS, ACT, and TUB2 trees. The EF-1a sequence of *Pyrenophora tritici-repentis* (GenBank No JQ314405.1) was utilized as the outgroup for the EF1-a tree. The find best model tool, implemented in MEGA11, was used to estimate the best genetic distance model for the construction of the phylogenetic trees. In total, 26 isolates for the ITS, ACT, and TUB2 barcodes and 19 for the EF-1a barcode were used to construct the individual phylogenetic trees. For each analyzed locus, the *Colletotrichum* isolates examined in our study were also aligned together with gene sequences of type isolates and other well-authenticated isolates of *Colletotrichum* species to facilitate comparison between them (Figure 6, Figure 7, Figure 8 and Figure 9).

The phylogenetic trees based on the barcode regions ITS, ACT, and TUB2 clearly clustered the isolates into three main clades with 100% bootstrap support: *C. acutatum*, *C. gloeosporioides*, and *C. coccodes.* Two subgroups can be distinguished in the *C. acutatum* clade based on the ITS, ACT, and TUB2 regions. The first one comprised the isolate from tomato CaD13-3, identified as *C. godetiae*, and the isolate Ca44-1 closely related to *C. salicis.* The second subgroup included Ca13-1, identified as *C. nymphaeae*, together with the reference strain CBS 490.92. Here, this strain clustered with *C. acutatum* samples and was identical with the *C. fioriniae* and *C. acutatum* (*s.s.*) strains. The four isolates were genetically well differentiated, which proves that each of them is actually a different species. For EF-1a, there were a limited number of sequences available in the GenBank that were homologous to this particular fragment of the *C. acutatum* isolates; therefore, it was not possible to determine the exact affiliation of *C. acutatum* strains based on these sequences.

The *C. coccodes* group was more heterogeneous, comprising isolates from different parts of various solanaceous crops. The ACT and TUB2 barcodes classified all isolates of the *C. coccodes* group as belonging to two main clusters based on used accessions from the NCBI GeneBank. One cluster contained isolates identical to *C. nigrum*, while the other one comprised isolates similar to *C. coccodes.* Furthermore, the TUB2 barcode enabled the differentiation of *C. nigrum* isolates into two sub-clusters. One sub-cluster contained the Bulgarian isolate Cc13-6, together with the three isolates from North Macedonia. The second sub-cluster included five isolates, mainly obtained from the fruits of different solanaceous hosts, with one exception: the isolate P13-2-1, which was obtained from potato stolon. The presence of both *C. nigrum* and *C. coccodes* NCBI accessions in the main *C. coccodes* cluster was observed for the ITS, tree showing that this barcode is not suitable for genetic discrimination of the studied *C. coccodes* isolates. The EF-1a barcode divided *C. coccodes* isolates into three sub-clusters. The CBS 527.77 strain and one isolate from potato CcP5-4 comprised the third one. Nevertheless, taxonomic assignment based on retrieved NCBI GenBank accessions showed them as belonging to the taxon *C. coccodes* only. This is probably due to the limited information available in the NCBI GenBank regarding this particular region of the *EF-1a* gene.

Within the gloeosporioides complex, the ITS, ACT, and TUB 2 barcodes separated the four isolates from pepper and tomato (CgB1-1, CgD6-1, CgD6-2, CgB27-1) into a subgroup different from that of the banana isolate Cm13, which was found to be identical to the *C. musae* strain. Here, they were classified as identical to *C. cigarro.*

**Figure A1:**
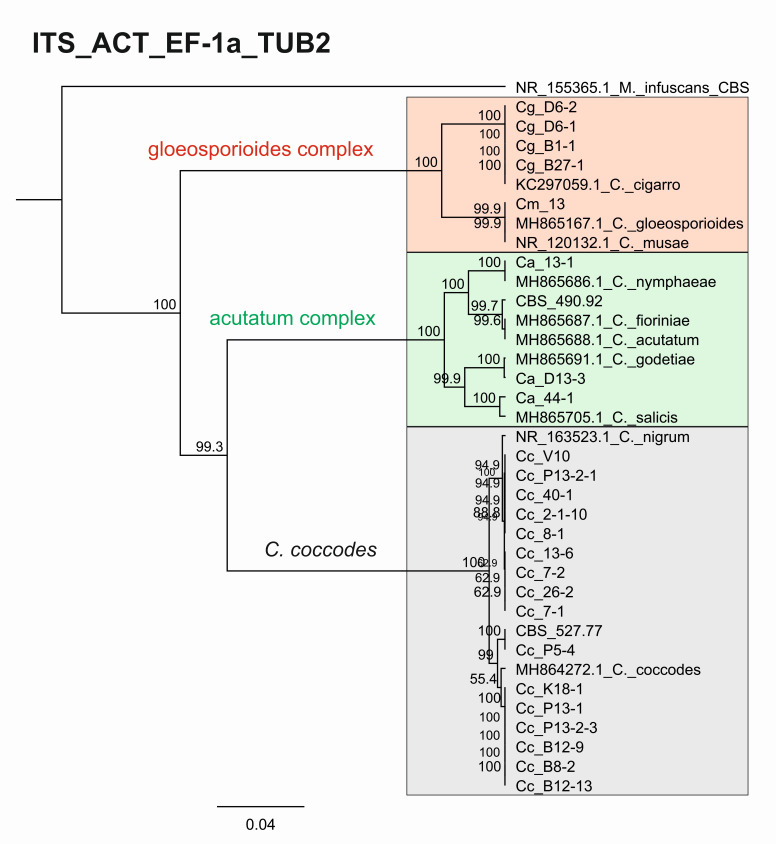
Consensus phylogenetic tree. Genetic diversity and taxonomic assignment of studied *Colletotrichum* isolates against accessions in the NCBI database and assessed by DNA barcodes for the concatenated loci ITS, ACT, EF-1a, and TUB2. The minimum and maximum lengths of non-reference sequences in the alignment were 536 nt and 1659 nt, respectively. The scale bar indicates the number of nucleotide substitutions per site as a parameter of phylogenetic distance. The *Colletotrichum* clades coccodes, gloeosporioides, and acutatum are shown by the grey, red, and green boxes, respectively.

The consensus tree based on the concatenated sequences of the four barcode regions ITS_ACT_EF_TUB entirely confirmed the species affiliations and phylogenetic relationships observed in the individual trees (Appendix A, Figure A1). The isolates were clustered into the three major clades, *C. acutatum*, *C. gloeosporioides*, and *C. coccodes*, with 100% bootstrap support. The four isolates belonging to the acutatum complex were clearly differentiated as different species, such as *C. godetiae* (isolate CaD13-3), *C. salicis* (Ca44-1), *C. nymphaeae* (Ca13-1), and *C. fioriniae* (reference strain CBS 490.92). The identification of the CgB1-1, CgD6-1, CgD6-2, CgB27-1 isolates as *C. cigarro* was also conclusively demonstrated. The *C. coccodes* isolates were divided into two subgroups—the first contained nine isolates identified as *C. nigrum* and the second included eight isolates identical to *C. coccodes*. Genetic variation within the respective groups of *C. coccodes* and *C. nigrum* isolates revealed by the individual trees was also evident from the consensus tree.

All sequences obtained in this study were submitted to GenBank, and the respective accession numbers for each isolate and locus are included in Appendix B, Table A1.

## 4. Discussion

The misidentification of *Colletotrichum* species and delineating taxa is a mistake frequently made, due to high sequence homogeneity, morphological uniformity within the genus, and the fact that there are few distinctive morphological characters available for identification [1]. The application of a polyphasic approach, including the analysis of geographical, ecological, morphological, and genetic data in order to establish a natural classification system for the genus *Colletotrichum* is highly recommended [8]. For this reason, multiple genes are required not just for elucidating the relationships between taxa but also for species identification. In this study, we performed a characterization of *Colletotrichum* isolates from different solanaceous crops in Bulgaria using DNA barcoding technology based on multiple genes, combined with classical morphological and pathogenicity assays.

The DNA barcodes used were efficient for the differentiation of *Colletotrichum* isolates at the species level, dividing them into three main clades: the acutatum complex, the gloeosporioides complex, and *C. coccodes*. However, the level of genetic discrimination between *Colletotrichum* specimens varied among the DNA barcode regions. TUB2, ACT, and EF-1α had better discrimination potential for the intra-species variation of *C. coccodes* isolates compared to the ITS region. Similarly, the ITS, ACT, and TUB 2 regions were efficient in distinguishing *C. acutatum* isolates at the species level.

Patterns of genetic diversity based on DNA barcodes reflect the morphological and cultural characteristics of *Colletotrichum* species. Data collected from phenotyping and from the available literature allowed us to make assumptions about the species affiliations of the analyzed *Colletotrichum* isolates. In this study, the isolates referred to as the acutatum species complex produced acervuli (asexual fruiting bodies) in the lesions on fruits. In culture, such structures were not observed, and conidia developed directly on the aerial mycelium of the colony. Only the isolate Ca44-1, obtained from a ripe tomato fruit in 2008, differed from the others in some morphological and cultural features and especially in the formation of a sexual morph in culture, along with conidia. Fertile structures of the sexual morph (perithecia, asci, and ascospores) have been found in older cultures or when grown in pairs in combination with the same or other isolates. Previously, *Glomerella miyabeana* was found to be closely phylogenetically related to *C. acutatum* [62], and it is capable of producing perithecia in nature. This fungal pathogen, which typically targets *Salix,* was known as the causal agent of willow black canker; however, in New Zealand, it has also been isolated from ripe fruit rots of tomato plants [62]. Moreover, the teleomorph of *C. acutatum* (*G. acutata*) was observed only in laboratory crosses of single-spore isolates [63]. These data led us to assume that our isolate Ca44-1 seemed to be *G. miyabeana* (unpublished data). Some years later, a detailed revision of the acutatum complex was made [37]. According to the results of the above study, only two species in the acutatum complex, *C. salicis* and *C. rhombiforme*, formed a sexual morph in culture. Ascospores of both species were almost the same size, but differed in shape. In addition, the conidia of *C. salicis* were smaller and had a regular cylindrical shape.

The multilocus phylogenetic analysis performed by Damm et al. (2012) [37], based on the sequences of the ITS region of rDNA and partial sequences of five other genes (ACT, TUB 2, CHS1, GAPDH, and HIS3), showed that the species *C. salicis* differed significantly from the others in the acutatum complex. This species was included in the list of currently accepted species of *Colletotrichum* [38]. *Glomerella salicis* (Fuckel) L. Hohm, *G. miyabeana* (Fukushi) Arx, and *G. lycopersici* F. Krüger are some of the synonyms of *C. salicis*. *G. lycopersici* was found as a pathogen on mummified fruits of *Solanum lycopersicum* (=*Lycopersicon esculentum*) in Germany (CBS 607.94) and its sexual morph has been identified as *Glomerella salicis* [37]. According to contemporary knowledge and based on morphological features (e.g., conidial shape and size; ascospore shape and size) and the cultural appearance (e.g., the amount and color of aerial mycelium; the time of perithecial production; the arrangement of perithecia across the culture), our isolate Ca44-1 was classified as *C. salicis*. This was further confirmed when our molecular data were completely identical to this species based on the ACT and TUB 2 secondary barcodes. The results presented for *C. salicis* show that taxonomic identification based on DNA barcodes is consistent with the phenotypic data.

The morphological and cultural characteristics of the two other studied isolates from the acutatum species complex (Ca13-1 and CaD13-3) were phenotypically similar, and it was difficult to identify them to the species level. A BLAST search and phylogenetic analysis of the ITS, ACT, and TUB2 genes revealed them as two different species: *C. nymphaeae* (Ca13-1) and *C. godetiae* (CaD13-3), thus supporting the findings of Damm et al. (2012) [37], and showing that the TUB2 and ACT sequences separated these species most effectively. *C. nymphaeae* and *C. godetiae* are among the currently accepted species of *Colletotrichum* under numbers 125 and 76, respectively [38]. The species *C. acutatum* was first reported in Bulgaria as the causal agent of strawberry anthracnose in 2002 [48]. The establishment of *C. acutatum* as a species complex and a detailed further revision led to the identification of 31 species, of which 21 were not previously recognized [37]. To date, 41 species have been described [64]. Three strawberry isolates, identified by Bobev et al. (2002) [48] as *C. acutatum* and deposited in the CBS, were included in the multilocus study of Damm et al. (2012) [37] and, according to the phylogenetic analysis, they were designated as one of the new species of the acutatum complex: *C. nymphaeae*. The first report of the occurrence of *C. acutatum* on peppers and tomatoes in Bulgaria was made in 2008 [49]. This identification was performed based on morphological and cultural characteristics, as well as on the ITS region, using the *C. acutatum*-specific primer CaInt2. It is reasonable to assume that the pepper and tomato isolates obtained from these authors could also belong to *C. nymphaeae*, as the phenotypic characteristics of the strawberry, pepper, and tomato isolates were very similar [48,49]. *C. nymphaeae* has also been reported to be the causal agent of anthracnose fruit rot in peppers (*Capsicum annuum*) in other parts of the world [65]. *C. acutatum* colonies had a chromogenic (pink) or nonchromogenic (white to gray) phenotype [7,66]. The results of the current study showed no distinct differences in characteristics among the pepper and tomato isolates. Our isolates were nonchromogenic, with colonies light grey to grey in color, with centers that were lighter from above and dark grey on the reverse side, including Ca13-1, which was identified as *C. nymphaeae*. All isolates studied by Bobev and Jelev [48,49,67] were morphologically and culturally uniform, being beige to grey on the top and salmon-orange to pink on the bottom, regardless of the host or origin. The main aim of the above-mentioned authors was to study strawberry anthracnose. Their samples were collected almost exclusively from or adjacent to strawberry production fields in the most popular strawberry growing regions in Bulgaria, and seemed to be phenotypically and genetically homogeneous. The origin of our isolates was in vegetable production areas and pepper- and tomato-growing experimental fields. This could be the possible explanation for the phenotypic differences of our isolate compared to those of other authors. Förster and Adaskaveg (1999) [68] also found that almond isolates of *C. acutatum* have two phenotypes—gray and pink. In the summarized information for *Colletotrichum* species from many countries on different continents, pepper isolates fell into group A2 and formed grey colonies [69].

The tomato strain CBS 490.92 (*C. fioriniae*), included as a reference in the *C. acutatum* group, differed significantly from our isolates in terms of the colony color, being saffron with beige to grey spots from above, and salmon, pale vinaceous, and olivaceous to purplish-grey on the reverse. This species has been not found in Bulgaria yet. In recent years, *C. fioriniae* has been reported in China as a fruit rot pathogen in *C. annuum* and *Capsicum* species [70].

The gloeosporioides species complex is the largest one within the genus *Colletotrichum* [38,71]. The name *C. gloeosporioides* is used in a broad (*sensu lato*) and in a narrow (*sensu stricto*) sense [23]. In a broad sense, it covers the entire complex and comprises 52 closely related species, including *C. musae* and *C. kahawae*, which form the two main clades in the complex bearing the same names [38]. The clade *C. kahawae* was found to consist of two subspecies: subspecies *kahawae* and subspecies *ciggaro*. The latter has been proposed as a new, genetically distinct subspecies by Weir et al. (2012) [23]. These two subspecies can be distinguished from all other species using only ITS sequences. The C. *kahawae* subspecies *kahawae* was known only from coffee (*Coffea* sp.) in Africa [72]. The *C. kahawae* subspecies *cigarro* has been reported in Australia, New Zealand, Europe, South Africa, Brazil, Colombia, and the United States through a number of hosts: *Areca catechu* L., *Banksia* sp., *Citrus reticulata* Blanco, *Dryandra* sp., *Dryas octopetala* L., *Hypericum perforatum* L., *Kunzea ericoides* (A. Rich.) Joy Thomps., *Leucospermum* sp., *Mangifera indica* L., *Miconia* sp., *Olea europaea* L., *Persea americana* Mill., *Rubus glaucus* Benth., and *Vaccinium* species [23,73,74,75,76,77]. In the gloeosporioides complex, several species were characterized by the formation of perithecia in culture—four species in the *musae* clade (*C. alienum*, *C. fructicola*, *C. queenslandicum*, and *C. salsolae*) and three in the *kahawae* clade (*C. clidemiae*, *C. kahawae* subspecies *ciggaro*, and *C. ti*) [23]. The size and shape of ascospores can also be utilized as a useful diagnostic feature at the species level. With the exception of *C. clidemiae* and *C. ti*, in most species, including *C. kahawae* subspecies *cigarro*, ascospores are strongly curved and usually tapered towards the edges. Recently, the *C. kahawae* subspecies *kahawae* and the *C. kahawae* subspecies *cigarro* have been recognized as distinct species, namely *C. kahawae* and *C. cigarro* comb. et stat. nov. [78].

Four isolates from pepper and tomato fruits included in the present study were previously identified as *C. gloeosporioides s.l.* based on molecular data for the ITS region alone, using the species-specific primer CgInt [45]. They were characterized by the simultaneous development of asexual and sexual morphs in culture. Along with the conidia, fertile perithecia were abundant, with a large number of ascospores, which were hyaline, unicellular, slightly curved or sickle-shaped, and slightly narrowed at the edges. Due to this peculiarity of the species, it was originally identified as an unusual *Colletotrichum* species from the group of *C. gloeosporioides* [45]. Our multilocus DNA barcoding analysis clearly showed the affiliation of these four isolates to the newly recognized species *C. cigarro* [23,78].

Our results indicate that the secondary barcodes, especially ACT and TUB2, have a high taxonomical resolution power for the isolates belonging to the *C. coccodes* group, dividing them into two major clades: *C. nigrum* and *C. coccodes*. *C. nigrum* comprised all isolates from the fruit rot of pepper, tomato, and eggplant, and the isolate P13-2-1 from potato stolon, which was phenotypically very similar. *C. coccodes* included six isolates from pepper and tomato roots and three from potato. The isolate P5-4 from potato tubers and the CBS 527.77 strain from tomato roots showed close similarities at both loci (ACT and TUB2), thus supporting morphological and cultural features. Other investigators [43] also distinguished these two clones in *C. coccodes* based on multigenic analysis (ITS, ACT, TUB2, CHS1, GAPDH). Both groups contained pepper and tomato isolates, but all potato isolates fell into the group of *C. coccodes*. Until recently, *C. nigrum* was considered suspicious [19], but now it is recognized as a singleton species with a close affinity to *C. coccodes* [38]. It is characterized by longer conidia and a greater length/width ratio [43]. A recent study of anthracnose on tomato fruit identified *C. nigrum* as the causative agent [79]. Phylogenetic analysis of the sequences of four loci (ACT, CHS-1, ITS, and TUB 2) showed 100% identity with the reference isolate of *C. nigrum*—CBS 169.49. The morphological and cultural signs and symptoms caused by the pathogen were very similar to those of *C. coccodes* [79].

Until the middle of the 20th century, *C. coccodes* was known to be the causal agent of fruit anthracnose, and another species, *C. atramentarium*, was associated with root rot in tomatoes and the black dot disease in potatoes. Some authors [80] supported the claim that the same fungus was the cause of root rot and anthracnose in tomato fruits, as well as potato disease. They recommended that only the name *C. coccodes* (Wallr.) be used. The same authors [81] found that an isolate of the fungus obtained from typical tomato root rot caused anthracnose on tomato fruits and tuber disease on potatoes. The results obtained in this study, as well as those of other contemporary authors [38,43,79], provide sufficient evidence suggesting the presence of two separate species (*C. coccodes* and *C. nigrum*).

The results of the pathogenicity tests showed that the fruits of pepper and tomato plants, the main vegetable crops in Bulgaria, were successfully infected with disease by all of the studied isolates, regardless of which host they were initially isolated from. The pathogenicity test on green apples ensured reliable results. One of the advantages of this test is the availability of fruit almost all year round, as well as its suitability to test several isolates on one fruit and the lower susceptibility to saprophytic invasion [51]. This assay contributed to the evaluation of the pathogenic properties of the isolates and, to some extent, to their differential diagnosis. The isolates of the *acutatum* and *gloeosporioides* groups caused the development of large necrotic spots, but with a different morphology. The lowest virulence of the *C. coccodes* isolates could be explained by a greater host specificity to some members of the Solanaceae family.

## 5. Conclusions

In conclusion, our study confirmed the applicability of morphological, cultural, and DNA sequence data to clarify relationships among the isolates studied. The obtained results add to previous reports about the relevance of DNA barcodes as useful markers for describing genetic diversity and species delimitation within the genus *Colletotrichum*. Our findings showed that three different species of the acutatum complex (*C. nymphaeae*, *C. godetiae*, and *C. salicis*) were associated with anthracnose fruit rot in peppers and tomatoes. The gloeosporioides complex was represented by four phenotypically and genotypically homogeneous isolates, defined as *C. cigarro*, and also related to fruit anthracnose of pepper and tomato plants. Applying ACT and TUB2 as secondary barcodes, the *coccodes* group was divided into two clades: *C. nigrum* and *C. coccodes*, showing good agreement with previous findings by other authors. *C. nigrum* was isolated predominantly from the fruits of the studied hosts, and *C. coccodes* was mainly isolated from the roots. In this study, only two *Colletotrichum* species (*C. salicis* and *C. cigarro*) produced sexual morphs in culture. The advantages demonstrated by the species *C. cigarro*, which has the ability to easily form a sexual morph and to attack a wide range of hosts, make it a threat to other hosts beyond the Solanaceae family. The species *C. godetiae, C. salicis*, and *C. cigarro* have not previously been reported as naturally occurring plant pathogens in Bulgaria. The identification of these species allows us to update the list of *Colletotrichum* species found in the region, and shows the extended geographical range of their distribution worldwide. Our results enrich the knowledge about the biodiversity and the specific features of the *Colletotrichum* species, which are pathogenic for the solanaceous hosts in Bulgaria, and serve as a scientific basis for efficient disease control and resistance breeding.

## Figures and Tables

**Table 1:** *Colletotrichum* isolates examined in the study.

Isolate Designation	Host/Substrate	Locality	Year of Isolation
Ca13-1	*Capsicum annuum*, fruit	Sofia, Bulgaria	2007
CaD13-3	*Solanum lycopersicum*, fruit	Sofia, Bulgaria	2013
Ca44-1	*Solanum lycopersicum*, fruit	Lozen, Bulgaria	2008
CBS490.92	*Solanum lycopersicum*	CBS	
CgB1-1	*Capsicum annuum*, fruit	Sofia, Bulgaria	2012
CgB27-1	*Capsicum annuum*, fruit	Sofia, Bulgaria	2012
CgD6-1	*Solanum lycopersicum*, fruit	Sofia, Bulgaria	2012
CgD6-2	*Solanum lycopersicum*, fruit	Sofia, Bulgaria	2012
Cm13	*Musa* sp., fruit	imported from Ecuador	2013
Cc2-1-10	*Solanum lycopersicum*, fruit	Sofia, Bulgaria	2010
CcB8-1	*Capsicum annuum*, fruit	Sofia, Bulgaria	2010
CcB8-2	*Capsicum annuum*, root	Sofia, Bulgaria	2011
Cc7-1	*Capsicum annuum*, fruit	Sveti Nikole, The Republic of North Macedonia	2011
Cc7-2	*Capsicum annuum*, fruit	Sveti Nikole, The Republic of North Macedonia	2011
Cc26-2	*Capsicum annuum*, fruit	Strumitsa, The Republic of North Macedonia	2011
CcB12-9	*Capsicum annuum*, root	Sofia, Bulgaria	2012
CcB12-13	*Capsicum annuum*, root	Sofia, Bulgaria	2012
CcK18-1	*Capsicum annuum*, root	Sofia, Bulgaria	2012
Cc13-6	*Capsicum annuum*, fruit	Sofia, Bulgaria	2013
CcV10	*Capsicum annuum*, fruit	Sofia, Bulgaria	2014
Cc40-1	*Solanum melongenae*, fruit	Sofia, Bulgaria	2009
P5-4	*Solanum tuberosum*, tuber	Kyustendil, Bulgaria	2013
P13-1	*Solanum tuberosum*, root	Lozen, Bulgaria	2013
P13-2-1	*Solanum tuberosum*, stolon	Sofia, Bulgaria	2013
P13-2-3	*Solanum tuberosum*, stem basis	Sofia, Bulgaria	2013
CBS 527.77	*Solanum lycopersicum*, root	CBS (Bulgaria)	1977

**Table 2:** Primer sequences for barcode loci amplified in this study.

Barcode Region	Primer Name	Primer Sequences 5′-3′	T ann	Reference
ITS	ITS1	TCCGTAGGTGAACCTGCGG	56 °C	[53]
	ITS4	TCCTCCGCTTATTGATATGC		
ACT	ACT-512F	ATGTGCAAGGCCGGTTTCGC	59 °C	[54]
	ACT-783R	TACGAGTCCTTCTGGCCCAT		
*EF-1a*	EF1-728F	CATCGAGAAGTTCGAGAAGG	58 °C	[54]
	EF1-986R	TACTTGAAGGAACCCTTACC		
*Tub2*	T1	AACATGCGTGAGATTGTAAGT	60 °C	[55]
	T2	TAGTGACCCTTGGCCCAGTTG		

**Table 3:** Conidial characteristics of the *Colletotrichum* isolates.

Isolate Designation	Length	Width	Shape
Ca13-1	(8.1−) **10.8 ± 0.1** (−13.4)	(3.3−) **4.2 ± 0.1** (−5.5)	Cylindrical to cylindric–clavate, pointed at one end and rounded at the other
CaD13-3	(9.2−) **12.1 ± 0.1** (−15.8)	(3.6−) **4.7 ± 0.1** (−5.9)	Cylindrical to fusiform with both ends acute or one end round and one end slightly acute
Ca44-1	(12.0−) **15.5 ± 0.2** (−21.5)	(3.5−) **4.5 ± 0.1** (−5.8)	Cylindrical to clavate with one end round and one end acute to truncate
CBS 490.92	(11.1−) **14.5 ± 0.9** (19.2)	(3.3−) **4.4 ± 0.1** (−5.9)	Fusiform to cylindrical with both ends acute
CgB1-1	(12.8−) **15.5 ± 1.1** (−17.6)	(3.7−) **4.7 ± 0.4** (−6.2)	Cylindrical with two to seven oil globules, rounded at both ends or pointed at one end and rounded at the other
CgB27-1	(12.5−) **15.1 ± 0.9** (−16.7)	(3.5−) **4.6 ± 0.3** (−6.3)	
CgD6-1	(13.0−) **15.8 ± 0.7** (−17.8)	(3.9−) **4.9 ± 0.4** (−6.5)	
CgD6-2	(12.6−) **14.9 ± 0.8** (−16.6)	(3.4−) **4.5 ± 0.5** (−6.1)	
Cm13	(11.4−) **13.3 ± 0.2** (−16.1)	(5.4−) **6.1 ± 0.1** (−7.1)	Cylindrical, with rounded ends
Cc2-1-10	(11.3−) **16.3 ± 0.2** (−20.6)	(2.9−) **4.6 ± 0.1** (−6.8)	Cylindrical, rounded at both ends
CcB8-1	(19.1−) **21.3 ± 1.7** (−24.6)	(3.1−) **4.1 ± 0.4** (−4.7)	Cylindrical, rounded at both ends
CcB8-2	(11.8−) **20.1 ± 0.1** (−21.6)	(4.0−) **5.1 ± 0,1** (−6.4)	Fusiform to cylindrical
Cc7-1	(9.4−) **15.8 ± 1.0** (−25.7)	(3.4−) **4.2 ± 0.1** (−5.0)	Cylindrical, rounded at both ends with two to seven globules
Cc7-2	(8.0−) **13.7 ± 0.5** (−21.2)	(3.4−) **4.7 ± 0.1** (−5.9)	Cylindrical, rounded at both ends
Cc26-2	(10.8) **15.0 ± 0.6** (−19.2)	(3.6−) **4.7 ± 0.2** (−5.8)	Cylindrical, rounded at both ends
CcB12-9	(12.7−) **17.0 ± 0.7** (−18.6)	(4.5−) **5.2 ± 0.1** (−5.9)	Fusiform to cylindrical
CcB12-13	(15.8−) **20.2 ± 0.1** (−23.6)	(3.2−) **4.2 ± 0.1** (−5.9)	Fusiform, attenuated at the ends
CcK18-1	(15.4−) **19.7 ± 0.2** (−23.4)	(3.3−) **4.8 ± 0.1** (−6.8)	Fusiform, attenuated at the ends
Cc13-6	(10.8−) **18.7 ± 0,4** (−27.5)	(3.8−) **5.8 ± 0,1** (−8.6)	Cylindrical, rounded at both ends
CcV10	(9.8−) **17.7 ± 0.4** (−26.3)	(3.2−) **4.9 ± 0.1** (−6.5)	Cylindrical, rounded at both ends
Cc40-1	(11.1−) **16.9 ± 0.5** (−20.2)	(3.1−) **4.8 ± 0.1** (−6.7)	Cylindrical, rounded at both ends
P5-4	(16.0−) **20.7 ± 0.1** (−25.4)	(3.3−) **4.5 ± 0.1** (−5.6)	Cylindrical, rounded at both ends
P13-1	(17.7−) **21.0 ± 0.1** (−25.2)	(3.5−) **5.0 ± 0.1** (−6.5)	Fusiform to cylindrical
P13-2-1	(7.8−) **15.0 ± 0.4** (−21.0)	(3.4−) **5.1 ± 0.1** (−7.0)	Cusiform, attenuated at the ends
P13-2-3	(18.7−) **21.3 ± 0.1** (−25.4)	(3.7−) **5.0 ± 0.1** (−6.2)	Fusiform to cylindrical
CBS 527.77	(12.9−) **18.6 ± 0.3** (−23.2)	(2.8−) **4.3 ± 0.1** (−5.6)	Fusiform to cylindrical

**Table 4:** Efficiency of PCR amplification and sequencing for *Colletotrichum* specimens for four DNA barcode regions.

Barcode Region	N	Alignment Length (bp)	% AE	% SE
ITS	26	556	100	100
ACT	26	246	100	100
EF-1a	19	242	75.0	100
Tub2	26	750	100	100

N = samples with successful amplification, % AE = Percentage of amplification efficiency, % SE = percentage of sequencing efficiency (from the amplified barcodes).

**Table 5:** Statistical parameters of genetic diversity within the genus *Colletotrichum*, calculated in MEGA11. Estimates of average evolutionary divergence over all sequence pairs from the number of base substitutions per site are shown. The standard error estimate(s) were obtained by a bootstrap procedure (500 replicates). The rate variation among sites was modelled with a gamma distribution (shape parameter = 1). All ambiguous positions were removed for each sequence pair (pairwise deletion option).

DNA Barcode Region	N Taxa	Ns	C	V	Pi	S	Average Pairwise Distance/SE
ITS	46	556	434	115	68	47	0.051/0.006
ACT	52	246	127	119	85	34	0.191/0.020
EF-1a	25	242	25	128	85	43	0.397/0.053
Tub2	57	750	421	328	270	58	0.170/0.011

Ns = total number of sites, C = number of conserved sites, V = number of variable sites, Pi = number of parsimony informative sites, S = singleton sites.

## Data Availability

Not applicable.

## Appendix A

The consensus tree (Figure A1) was drawn using Geneious tree builder (Tamura-Nei genetic distance model, UPGMA tree building method, 1000 Bootstrap replicates, consensus method: majority greedy clustering) [82].

## Appendix B

**Table A1:** NCBI accession numbers of the DNA sequences identified in the current study.

**Isolate**	**ITS**	**ACT**	**EF-1a**	**TUB2**
Ca13-1	Acc. No OP587502	Acc. N xx-xx	Acc. N xx-xx	Acc. N xx-xx
CaD13-3	Acc. No OP587503	Sub2630242	Sub2630459	Sub2630466
Ca44-1	Acc. No OP587504			
CBS 490.92	Acc. No OP587505			
CgB1-1	Acc. No OP587506			
CgB27-1	Acc. No OP587507			
CgD6-1	Acc. No OP587508			
CgD6-2	Acc. No OP587509			
Cm13	Acc. No OP587510			
Cc2-1-10	Acc. No OP587511			
CcB8-1	Acc. No OP587512			
CcB8-2	Acc. No OP587513			
Cc7-1	Acc. No OP587514			
Cc7-2	Acc. No OP587515			
Cc26-2	Acc. No OP587516			
CcB12-9	Acc. No OP587517			
CcB12-13	Acc. No OP587518			
CcK18-1	Acc. No OP587519			
Cc13-6	Acc. No OP587520			
CcV10	Acc. No OP587521			
Cc40-1	Acc. No OP587522			
P5-4	Acc. No OP587523			
P13-1	Acc. No OP587524			
P13-2-1	Acc. No OP587525			
P13-2-3	Acc. No OP587526			
